# Scanning a DNA Molecule for Bound Proteins Using Hybrid Magnetic and Optical Tweezers

**DOI:** 10.1371/journal.pone.0065329

**Published:** 2013-06-03

**Authors:** Marijn T. J. van Loenhout, Iwijn De Vlaminck, Benedetta Flebus, Johan F. den Blanken, Ludovit P. Zweifel, Koen M. Hooning, Jacob W. J. Kerssemakers, Cees Dekker

**Affiliations:** Department of Bionanoscience, Kavli Institute of Nanoscience, Delft University of Technology, Delft, The Netherlands; Swiss Federal Institute of Technology Zurich, Switzerland

## Abstract

The functional state of the genome is determined by its interactions with proteins that bind, modify, and move along the DNA. To determine the positions and binding strength of proteins localized on DNA we have developed a combined magnetic and optical tweezers apparatus that allows for both sensitive and label-free detection. A DNA loop, that acts as a scanning probe, is created by looping an optically trapped DNA tether around a DNA molecule that is held with magnetic tweezers. Upon scanning the loop along the λ-DNA molecule, EcoRI proteins were detected with ∼17 nm spatial resolution. An offset of 33±5 nm for the detected protein positions was found between back and forwards scans, corresponding to the size of the DNA loop and in agreement with theoretical estimates. At higher applied stretching forces, the scanning loop was able to remove bound proteins from the DNA, showing that the method is in principle also capable of measuring the binding strength of proteins to DNA with a force resolution of 0.1 pN/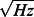. The use of magnetic tweezers in this assay allows the facile preparation of many single-molecule tethers, which can be scanned one after the other, while it also allows for direct control of the supercoiling state of the DNA molecule, making it uniquely suitable to address the effects of torque on protein-DNA interactions.

## Introduction

**Figure 1:**
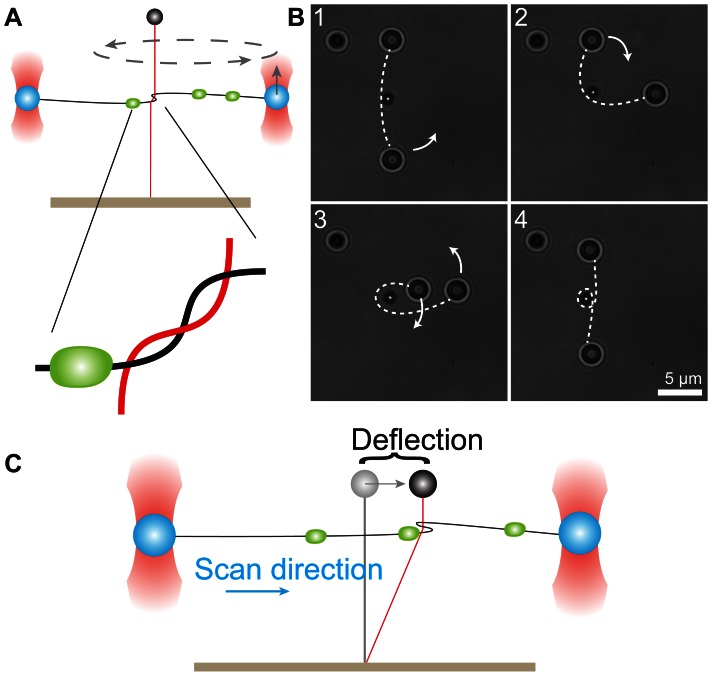
Detection of DNA-bound proteins using a scanning DNA loop and magnetic and optical tweezers. (A) A DNA loop is created by moving the optically trapped beads (blue) around the magnetic-bead DNA tether (red). Zoom in shows the intertwined geometry of the DNA loop. The green dot depicts a bound protein. (B) Top view image series showing the formation of the DNA loop. The loop is made by rotating the beads trapped by optical tweezers around the magnetic bead which is located in the center of the image. The position of the DNA molecule is indicated by the white dashed line. The bead in the upper left corner functions as a reference and is stuck to the surface of the flow cell. (C) The loop is scanned in the horizontal direction by moving both optically trapped beads in concert. Upon encountering a bound protein the DNA cannot slide through the loop anymore and the magnetic bead tether is deflected.

DNA is the center of action in cells: proteins bind to specific sequences, RNA-polymerases move along and transcribe genes, DNA is modified and wrapped around nucleosomes. Together the actions and locations of these proteins determine how genetic information is used in a cell [1]. There is thus an evident need for techniques which are able to localize DNA-bound proteins and probe their interactions. An array of single-molecule techniques, which allow for precise control and detection of individual DNA molecules and proteins, have made it possible to determine many of the intrinsic properties of DNA and associated proteins [2], [3]. Electron microscopy and AFM allow for the visualization of proteins on DNA, but require the immobilization of DNA and proteins on a surface [4]–[6]. Optical tweezers have been used to monitor the movement of single proteins along DNA in buffer, but rely on labeling of the proteins for optical or mechanical detection [7]. Inspired by the work of Noom et al. [8], we developed a method that allows label-free high-accuracy detection of proteins bound to DNA by the use of a scanning loop formed by one DNA molecule that is looped around another [9]. Figure 1A shows a simplified scheme depicting the loop formed between the two DNA molecules that are held in optical and magnetic tweezers (Fig. 1B). When the loop is scanned, by moving the horizontal DNA molecule sideways with optical tweezers, a DNA-bound protein will act as a friction barrier. Upon encountering a bound protein, the sliding loop will be halted and the magnetic bead will be displaced, thereby indicating the position of the protein (Fig. 1C).

The combined use of magnetic and optical tweezers to create and manipulate the DNA loop has several advantages over using only optical tweezers: it allows for the facile measurement of multiple molecules, it provide increased force resolution as magnetic tweezers operate at the thermal force limit, and it creates new possibilities to probe the influence of DNA supercoiling and its influence on DNA-protein interactions [10]. In this paper, we present the development of such a combined optical and magnetic tweezers for scanning a DNA molecule to probe its bound proteins. To demonstrate the functionality of this scanning technique, we used it here to accurately determine the position of EcoRI proteins bound to DNA. At low applied stretching forces, we observe that the loop gets stuck for a short period of time upon encountering a protein and then passes over. At higher stretching forces, the force exerted by the scanning loop on the protein can be used to displace the protein.

## Results

**Figure 2:**
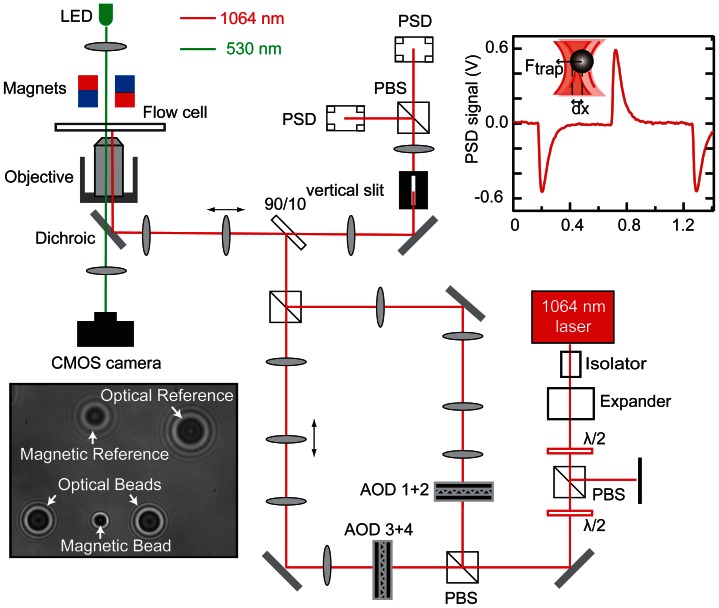
Schematic illustration of the hybrid magnetic and optical tweezers setup. A 4 W 1064 nm laser beam passes first trough a beam isolator and is expanded by a beam expander. Two half-wave plates (λ/2) and a polarizing beam splitter (PBS) are used to control the power in both polarizations of the beam. The beam is subsequently split and sent through a pair of acousto-optical-deflectors (AODs) to steer the beam. Lenses are used to further expand the beam and transfer the beam deflection from the conjugate plane of the AODs to the back focal plane of the objective. Two lenses are mounted on translation stages to allow adjustment of the position of the optical traps in the axial direction. 10% of the back reflected light is collected with a plate beam splitter and directed onto a separate position sensitive detector (PSD) for each trap. A vertical slit is used to block reflections from the surfaces of the flow cell and objective. Optical-trap stiffness was calibrated by using a square-wave method where the optical traps are quickly displaced and the return of the bead to the equilibrium position is monitored (see PSD signal top right). The positions of both optical beads, the magnetic bead and the flow cell surface was determined using video microscopy (bottom left image).

We designed and built a combined magnetic and dual-beam optical tweezers instrument that allows manipulation and detection of interaction forces via an optically trapped DNA molecule looped around a second DNA molecule that is tethered by magnetic tweezers (Fig. 2) [9]. Magnetic tweezers were used to create a vertical DNA tether between the flow cell surface and a magnetic bead by a pair of magnets positioned above the flow cell [11]. Two optical traps were generated by splitting a beam into two orthogonally polarized beams, which could be independently steered in *x*- and *y*-direction by acousto-optical deflectors (AODs). The microsecond-response time of the AODs was used to calibrate the stiffness of the optical-traps by quickly displacing the trap position and monitoring the response of the bead via detection of backscattered light [12], [13]. In this detection configuration, surface reflections from the flow cell, that are of the same order of magnitude as the backscattered light from the trapped bead, can reduce the signal-to-noise ratio (SNR). We were able to substantially improve the SNR by attenuating the background reflections well below the bead signal by spatially filtering these with a slit positioned in the detection path. The slit, however, limits the detection via backscattered light to one dimension, as reflected light from displacements of the bead perpendicular to the slit will also be blocked. Generally, this is not a problem as either the *x*- or *y*-dimension is used for force measurements in an experiment. Interaction experiments involving two molecules require the simultaneous detection of all beads and their relative positions. Video microscopy was used to monitor the position of both the magnetic and optically trapped beads with nanometer resolution in three dimensions [14], [15]. The position of the flow cell surface was tracked via a reference bead immobilized on the surface. The stretching force acting on the magnetic bead was determined from the bead’s thermal fluctuations, as measured by video microscopy. The forces acting on the optically trapped bead tether were obtained by using video microscopy to determine bead displacements from their respective trap center positions. The previously calibrated trap stiffness was subsequently used to convert these bead displacements to forces.

A laminar flow cell with multiple parallel flows was used to create separated buffer environments and enable the step-by-step assembly of DNA tethers between the two beads of the dual optical trap [9], [16], [17]. A stable flow pattern was created by providing gas pressure to all flow channels from a shared regulated pressure chamber. All channels were switchable by valves and the relative flow rates were set by the flow resistance of the connecting tubing, resulting in total flow rates of approximately 5 ml/hour for the flow cell. The flow cell was mounted on a translation stage to enable controlled movement of the optically trapped beads into each of the different laminar flow channels.

**Figure 3:**
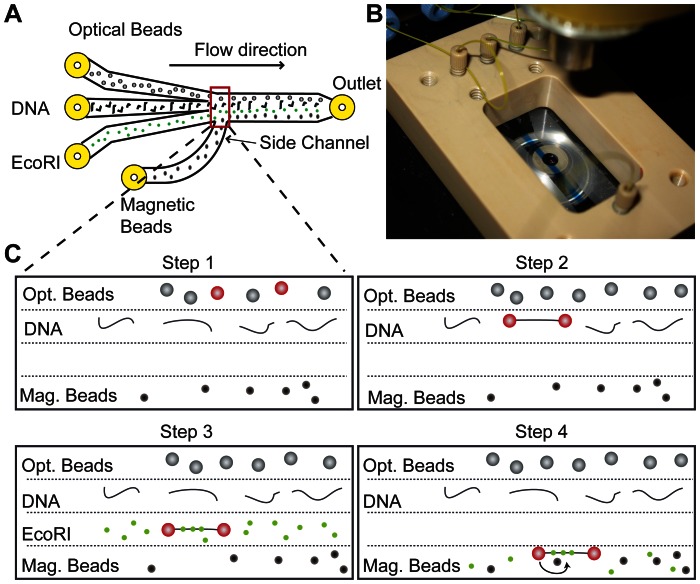
Step by step assembly of DNA tethers using a laminar flow system. (A) Schematic of the laminar flow cell showing four different inlets, that are combined into a central channel. Magnetic beads are flushed in via a side channel that exits perpendicular to the main flow direction. This allows for a very low flow rate (<10 µL/h) during experiments, while preventing diffusion into this channel. (B) Photograph of a flow cell using three channels, the central channel contains blue dye, showing clear separation by laminar flow from the other two flow lanes. (C) Illustration of the step-by-step assembly procedure to perform the scanning loop experiments. In step 1 two beads are caught by optical tweezers. In step 2 a biotin-labeled DNA molecule is tethered between the streptavidin-coated beads. In step 3 the presence of a single DNA molecule is confirmed by force extension analysis and EcoRI proteins are allowed to bind to the tethered DNA molecule. In step 4 the DNA tether is brought within close distance of a magnetic bead tether and a loop is formed. A low concentration of EcoRI proteins is present in this channel to assure that proteins remain bound to the DNA.

Each experiment starts by introducing magnetic beads into the flow cell and tethering these via a single DNA molecule to the surface in a side channel of the flow cell. This procedure creates many (>100) DNA tethers and is similar to the procedure commonly used in magnetic tweezers experiments [11]. The side channel, that exits perpendicular to the main flow channel, allows for near-zero flow rates around the magnetic-bead tethers while preventing mixing with the other buffer flows. To create a single-molecule DNA tether between the optically trapped beads a step-by-step assembly procedure was employed, (Fig. 3). First, two streptavidin-functionalized polystyrene beads are caught in two optical traps (Fig. 3C). Next, these beads are moved to a channel containing biotin end-labeled λ-DNA molecules, 16 µm (48 kb) in length. The formation of a DNA tether between the beads is monitored by moving one bead to and from the other bead until a force on the stationary bead is detected. Subsequently, the two-bead DNA tether is moved to a channel without DNA and its force-extension response is probed to verify the presence of a single DNA molecule. Finally, interaction experiments are started by moving the optically trapped molecule within close proximity of the magnetic bead tether and creating a loop by moving one of the optically trapped beads around the magnetic DNA tether (Fig. 1B).

The DNA loop acts as a scanning probe, enabling the label-free detection of DNA-bound proteins. After making a loop around a 4 µm (12 kb) long magnetic bead tether, as illustrated in Fig. 1A–B, we position the optically trapped beads in line with the magnetic bead, but at a lower z-position, thus creating a crossed DNA configuration at a height approximately halfway between the magnetic bead and flow cell surface, i.e. 2 µm below the bead. The precise geometry of the DNA at the loop (c.f. Fig. 1A zoom in) is set by the bending stiffness of the DNA and the tensions applied on the magnetic and optically trapped DNA molecules. Unless otherwise stated the tension in both molecules in the experiments reported below was set in the range of 12–16pN to create a symmetric structure.

The displacement of the magnetic bead acts as a very sensitive probe for the interactions between the two DNA molecules. Any friction that is encountered by the sliding loop while scanning will displace the magnetic bead tether, and consequently the magnetic bead, sideways (Fig. 1C). In the absence of DNA-binding proteins, we observed no interactions while scanning the λ-DNA molecule. This indicates the absence of friction between the DNA molecules even at these rather high forces, as also observed previously [8], [9], [18]. This frictionless sliding is likely due to the electrostatic repulsion between the negatively charged DNA backbones, which prevents direct mechanical contact [19].

**Figure 4:**
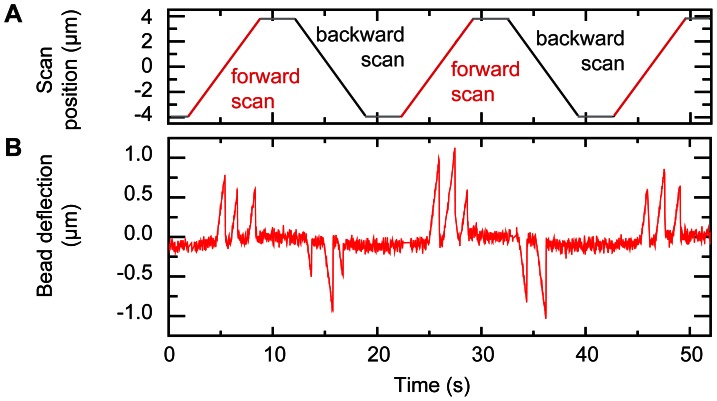
DNA-bound proteins are detected by scanning a DNA loop. (A) Scan position defined as the mean position of the two optically trapped beads (red lines are forward scans, black lines are backward scans). (B) Magnetic bead deflection showing three spikes in both the forward and backward scans indicating the presence of DNA-bound proteins. The tension in both the magnetic bead tether and optical bead tether was set at 12 pN.

To demonstrate the ability of the setup to detect proteins along a DNA molecule, we performed measurements in the presence of the restriction enzyme EcoRI under noncleaving conditions. The EcoRI restriction enzyme binds specifically to its recognition sequence GCTT but does not cleave the DNA in the presence of Ca^2+^ ions [20]. The λ-DNA molecule, that is bridging the two beads in the dual-trap optical tweezers, contains five recognition sites along its length that function as site-specific markers to validate the detection method. Figure 4 shows an example of two consecutive forward and backward scans of a λ-DNA molecule in the presence of 50 nM EcoRI, made by horizontally moving the optically trapped beads at a scan rate of 1 µm/s. We clearly identify three spikes in both the forward and backward scans. The other two EcoRI positions were located outside of the scan range and were therefore not detected. The observed bead displacements result when the scanning loop encounters a protein, which temporarily blocks the further traversal of the loop until the protein and the loop slip across each other. The displacements were present in both the forward and the backward scans. Occasionally, we observed spikes at other locations along the DNA, which most likely correspond to nonspecifically bound proteins along the DNA. These events however most often disappeared after a single scan (data not shown), indicating that the proteins had dissociated from the DNA.

**Figure 5:**
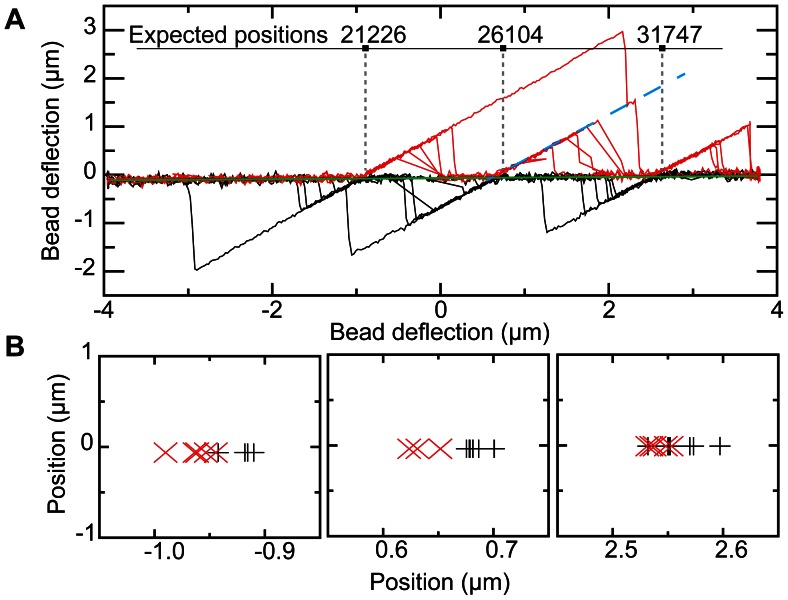
Detection of DNA-bound EcoRI proteins. (A) 7 consecutive scans superimposed (red lines forward scans, black lines backward scans, data from a single molecule is shown). Protein positions (grey dashed lines) were determined from the intersection of linear fits to the baseline (green line) and individual identified spikes (blue dashed line). Expected positions based on the DNA sequence are indicated at the top, numbers indicate sequence position in bases. (B) Protein position determined from the forward (red crosses) and backward scans (black crosses) of panel A. EcoRI positions are systematically detected slightly to the left in forward scans compared to the positions detected in the backward scans.

Figure 5A shows superimposed experimental data from 7 consecutive forward and backward scans. This plot clearly shows that the spikes originate from three locations on the λ-DNA molecule, at positions that very closely match the positions expected from the DNA sequence. To measure the positions of the bound proteins, we calculated the intersection point between the baseline and linear fits to individual spikes (Fig. 5A, green line and blue dashed line, respectively). From the standard deviation of repeat measurements on the same protein we estimate the spatial resolution to be ∼17 nm or ∼50 bp (6 different DNA molecules were measured and 88 protein positions were fit). Comparison of the known distances between the specific binding locations of the EcoRI protein to the measured positions yielded an average error of 15±4 nm (s.e.m. *n* = 64) for the relative position accuracy. The main source of error in these measurements are the thermal fluctuations of the magnetic bead, that are not averaged out within the finite time of a protein detection event. The absolute position of the bound proteins was calculated as the mean of the detected positions with respect to the center of the DNA molecule. The standard deviation of these absolute positions was 82 nm. This larger error likely results from differences in size of the optically trapped beads that directly affect the calculated center position of the DNA molecule. The typical 5% size variance of the used beads, i.e. 105 nm for the 2.1 µm beads, indeed agrees with the observed positional error.

Surprisingly, we found that there was a consistent offset of the positions detected in forward and backward scans. Figure 5B shows a separate analysis of forward (red points) and backward (black points) for the data shown in Figure 5A. There was a consistent offset of 33±5 nm, (s.e.m. *n = *18) between the detected positions in forward and backward scans. This may be understood by considering the geometry of the scanning loop (Fig. 1A zoom in). The probe DNA will start to displace the magnetic bead tether as soon as a protein encounters the loop, i.e. the detected position will not be in the middle of the DNA loop but at the point where the protein first encounters the loop. For scans in the reverse direction the detected interaction point will be at the other side of the scanning loop. The offset between forward and reverse scans is therefore a measure of the size of the probing loop. We can estimate the size of the loop by a simple model based on the mechanical properties of the DNA. Assuming a circular shape of the loop, its diameter will be: 
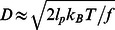
, where 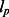 is the persistence length of DNA of about 50 nm and, *f*, the applied tension [3]. This gives a loop diameter of 5–7 nm for tensions in the range of 10–20 pN. The total length of DNA in the loop will then be 16–22 nm. Accounting for the size of the EcoRI protein of ≈ 3 nm, we thus expect an offset of 19–25 nm, which is of similar size as the experimentally determined value of 33±5 nm. The slightly larger measured loop size may result from the fact that the experimental DNA geometry likely differs substantially form an ideal circular loop (Fig. 1A zoom in).

**Figure 6:**
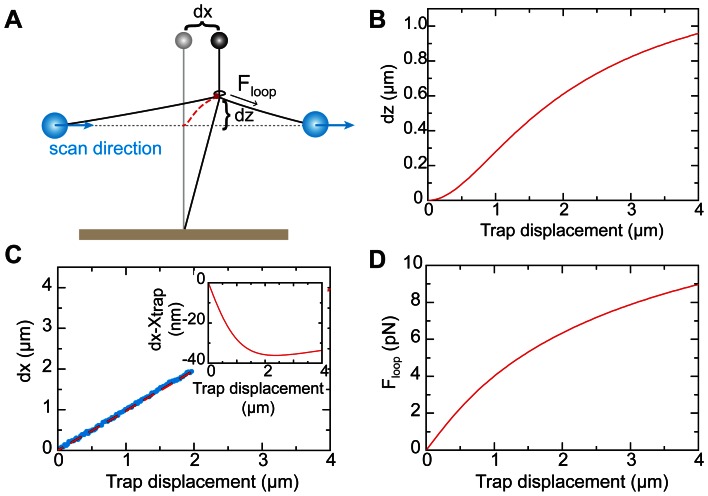
Calculated position and forces acting on the scanning DNA loop. (A) schematic diagram, not to scale, showing the displacement of the DNA loop as the optically trapped beads are scanned to the right. Forces and positions were calculated iteratively for a 48 kb DNA molecule, at an initial loop height of 2 µm above the surface, initial tensions were 10 pN for both DNA molecules, and optical trap stiffness was set to 100 pN/µm. The elasticity of the DNA was modeled by the worm-like-chain model. As the loop encounters a bound protein or other obstacle during the scan, the DNA will no longer slide through the loop but displace the magnetic bead by *dx*. Due to the direction of forces acting on the loop it will move to the right and upward following the track depicted by the red dotted line. (B) Calculated upward movement, *dz*, of the loop as a function of trap displacement after encountering a protein. (C) Movement of the magnetic bead in the scan direction after the loop encounters a bound protein (blue points experimental data, red dashed line calculation). The displacement of the magnetic bead is almost equal to that of the scanning traps, inset shows the difference between the magnetic bead position, *dx*, and trap position, *Xtrap;* note the nm scale. (D) Calculated force acting on a protein blocking the DNA loop.

How large is the force acting on the DNA loop, that leads to the displacement of the magnetic bead? We calculated the positions of the loop and the magnetic bead for different scan positions, see Fig. 6. The loop was considered freely sliding along the DNA, but the protein was not allowed to pass through the loop. At each position the forces acting on the loop, i.e. those of the two DNA molecules and their geometry (Fig. S1–S3) were solved iteratively and the stretching of the DNA molecules was accounted for by the worm-like-chain model [21]. The calculations show that the local geometry of the forces pushes the loop upward as the magnetic bead is displaced (Fig. 6A–B, and S2). The experimentally observed near linear displacement of the magnetic bead with scan position was accurately reproduced by the numerical calculations, as shown by the overlap of the experimental data and calculation results (Fig. 6C, blue points, and red dashed line respectively). For very large bead displacements the calculated force acting on the loop increase sublinear (Fig 6D). For small displacements, the force scales nearly linear with magnetic-bead displacement with a stiffness of 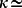
5 pN/µm, making the magnetic bead a sensitive probe for the exerted force. We can now calculate the smallest detectable force of the scanning method as 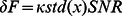, where *std(x)* is the sampling-time dependent standard deviation in bead positions and *SNR* is the desired signal-to-noise ratio. Setting *SNR = *4 for a 95% confidence bound and using the standard deviation of bead position σ = 0.05 µm at 50 *Hz* the force detection limit becomes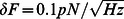.

The force exerted by the loop acts along the direction of the DNA, mimicking the cellular forces generated by motor proteins *in vivo*. To determine if these applied forces could dislodge the bound EcoRI proteins, experiments were performed at different forces. At moderate stretching forces of ∼12 pN for both the magnetic and optical tethers, proteins generally remained bound, and were observed for many consecutive scans (see Fig. 4). At forces above 20 pN, applied to both the magnetic and optical tweezers tethers, proteins were, however, easily dislodged and disappeared after a single or several scans (Fig. S4).

## Discussion

We have developed a new method which combines magnetic and optical tweezers to localize DNA-bound proteins. The use of magnetic tweezers in this assay presents several advantages compared to the elegant method using 4 optical traps to hold two DNA molecules, described previously by Dame et al. [22] and Noom et al. [8]. The 4 optical trap method is not easily adapted to tether two DNA molecules of different sequence or length, as bead-DNA constructs must be prepared simultaneously in the same flow. This limits the ability to address DNA-sequence-related biological questions with this approach. It is also an experimentally complex method, which is likely responsible for its limited use. The combination of magnetic tweezers in addition to optical tweezers, however, creates a robust platform to prepare a large number (>100) of tethered DNA molecules in advance. Multiple molecules can thereafter be scanned sequentially, without having to capture new beads with optical tweezers. The combination of magnetic and optical tweezers also facilitates the use of different DNA molecules, as the two DNA tethers are assembled independently. Importantly, magnetic tweezers allow to apply torque on the tethered DNA molecule and our method can thus be extended to induce supercoiling and probe the binding affinity of proteins in response to torque. Finally, magnetic tweezers are capable of detecting very low forces (down to ∼10 fN) as their detection limit is principally set by thermal noise.

Several other techniques have been developed to localize DNA-bound proteins. EM allows for direct visualization of proteins and DNA, but requires the samples to be frozen or deposited on a surface [23]. The best established scanning probe technique is atomic force microscopy (AFM), which uses a scanning tip to image or manipulate DNA and proteins immobilized on a surface [5], [6]. In contrast to AFM, the method described here holds the DNA molecules free in solution and away from the (typically highly charged) surface. Importantly the forces applied by the scanning DNA molecule act along the DNA contour, closely resembling *in vivo* processes, where motor proteins move along and remodel DNA-bound proteins. More recently solid-state nanopores have also been used to identify local structures on DNA molecules [24]. These rely on the detection of a change in ionic current as a DNA molecule passes through a nanometer-sized hole and partially blocks it. Nanopores offer in principle straightforward operation, but the high speed of translocation makes it difficult to detect individual bound proteins, and the exerted force is ill defined [25].

The experiments described here used a horizontal scanning configuration, where the optically trapped DNA molecule was moved from side-to-side. This is however not the only mode of operation, as alternatively the optical trapped tether can also be displaced vertically to scan along the magnetically trapped DNA molecule. Unlike the horizontal scanning procedure, however, this method is asymmetric since one side of the DNA molecule is attached to surface and the other to the magnetic bead. If the loop is scanned in the upward direction the applied force upon encountering a bound protein will increase gradually due to the stretching of the DNA between the flow cell surface and the scanning loop. If the loop is scanned towards the surface and encounters a DNA-bound protein it will pull the magnetic bead down. The force applied to the loop will, in this case, be nearly constant and equal to the force applied on the magnetic bead. This scanning direction therefore provides a simple method to apply constant force on DNA-bound proteins and monitor their stability. Many biological processes rely on proteins diffusing or actively moving along DNA. These moving proteins may encounter DNA-bound proteins that can stop or slow their progress. The label-free properties of the proposed method and the ability to use the DNA loop as a controlled roadblock will allow the study of these poorly understood interactions. We envision that the developed method using a scanning-DNA loop and the combination of magnetic and optical tweezers will not only prove useful for the study static bound proteins, but can also be used to count or track moving proteins.

## Materials and Methods

### Hybrid Magnetic and Optical Tweezers Setup

A custom dual-beam optical tweezers and magnetic tweezers instrument was used to perform the scanning experiments. Figure 2 shows a schematic outline of the setup. The optical traps are generated with a 1,064 nm laser (diode-pumped, solid-state Nd:YAG, Coherent Compass 1064–4000 M), that is isolated against back-reflections by a Faraday isolator. A half-wave plate and a polarizing beam splitter allow adjustment of the total laser power. A second half-wave plate controls the polarization of the beam and the power in each of two independent traps formed after splitting the beam on the basis of polarization. Two-axis acousto-optical deflectors (AODs, DTSXY-250, AA Opto-Electronic) enable independent steering of the both traps. The RF-frequency electrical drive signals for the AODs were generated using a home-built four channel Direct Digital Synthesizer (DDS, AD9959, Analog Devices) and subsequently amplified to 1 W (ZHL-5W-1, mini-circuits) [26]. A field programmable gate array (FPGA, NI PXI-7853R) is used to interface with the DDS as well as digitize and process the optical detection signals from position sensitive detectors (PSDs). The combination of DDS and FPGA allow for fast beam steering as well as position feedback [27]. In both beam paths, we implemented two telescope systems, a 1∶1 followed by a 2∶3 telescope, before recombining both beams with a polarizing beam splitter cube. In one of the paths, the first lens in the 2∶3 telescope system could be displaced axially, using a computer-controlled translation stage (8MT167–25, Standa), to move that trap in axial direction. A final telescope, equipped with a translation stage (8MT167–25, Standa), controls the axial position of both traps and expands the beam before coupling into a water-immersion objective (Nikon CFI PLAN APO VC 60X WI). The magnetic-tweezers magnets, positioned above the flow cell, make transmission-based detection of the laser beam impossible. Instead, detection of the optically trapped beads is performed by detecting the back-reflected signal [12]. Backscattered light is collected by the objective and separated using a 90–10 plate beam splitter and directed onto a position-sensitive detector (PSD, DL100-7PCBA3, Pacific Silicon Sensor). This high-bandwidth detector allows performing trap-stiffness calibrations both via a power-spectrum analysis as well as displacement methods [13], [28], [29]. The magnetic tweezers consist of an external magnet placed above the flow cell. External motors (M-126.PD2 and M-150.PD, Physik Instrumente) allow positioning and rotating the external magnets thereby stretching and twisting the magnetic-bead DNA tethers. Positional tracking of two optically trapped polystyrene beads (streptavidin coated, 2.1 µm, Spherotech), the superparamagnetic bead (streptavidin coated, 1 µm, Dynabeads MyOne, Invitrogen), and two fiducial markers (one superparamagnetic bead and one polystyrene bead placed on the bottom of the flow-cell) was performed using video-microscopy (at 50 Hz or 100 Hz). The forces applied on the optically-trapped beads were extracted from the position difference between the set trap position and those detected by video-microscopy using the trap stiffness determined from calibrations using the back-reflected light.

### Buffers and DNA Constructs

All measurements were carried out at 22 °C and were performed in a buffer of 10 mM Tris (pH 7.4), 100 mM NaCl, 0.125 mM CaCl_2_.

#### λ-DNA 48 kB construct

The 12 base pair overhangs on the λ-DNA are filled by a Klenow polymerase for 2 h at 37°using biotin-labeled cytosines and regular ATP, GTP and TTP nucleotides. The construct was purified by phenol extraction, ethanol precipitated and the pellet was finally dissolved in 10 mM Tris (pH 8.0), 1mM EDTA, 1% ethanol.

#### DNA magnetic tweezers construct

A 12 kb magnetic tweezers construct was prepared by PCR and subsequent ligation of a digoxygenin labeled handle as follows: A 11940 kb fragment was prepared by PCR on a λ-DNA template. A 1238 bp digoxigenin-labeled fragment was prepared by PCR on a pbluescriptIISK+ template a standard PCR reactions was performed, except 2 µl of digoxigenin-11-2′-deoxy-uridine-5′- triphosphate (dig-dUTP, Roche) is added. PCR products were purified with nucleospin extract II kit (Machery Nagel). Both fragments were cut with XhoI restriction enzyme giving to 2 fragments after digestion (554 and 684 bp) for the digoxygenin labeled handle and a 11926 bp fragment from the λ-DNA pcr. The digestions were purified with a nucleospin extract II kit (Machery Nagel), mixed and ligated with T4 DNA ligase the labeled ends were added in 10 molar excess. To purify the construct the ligation was phenol extracted and ethanol precipitated. The pellet is dissolved in 10 mM Tris (pH 8.0), 1 mM EDTA, 1% ethanol.

## Supporting Information

Figure S1
**Force diagrams for the free-sliding (A) and non-sliding (B) case.** Force balances at the loops shown in Figure S1 were iteratively solved using the following equations, 
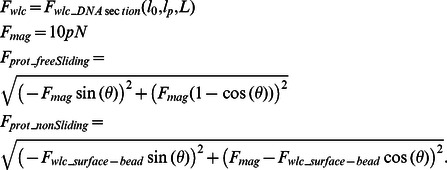
where *F_wlc_* was calculated using Ref. 21, with *l_0_* the contour length of a DNA section, i.e. the contour length of a section of DNA spanning from bead to loop, *l_p_* the persistence length of DNA = 50 nm, and *L* the length of a DNA section. *F_mag_* = 10 pN was the force applied on the magnetic bead and *F_prot_freeSliding_* and *F_prot_nonSliding_* represent the forces acting on the protein/loop for the free-sliding and non-sliding case respectively.(TIF)

Figure S2 **Calculated position and forces acting on the scanning DNA loop for the free-sliding case.** (A) Loop x-position and (B) loop z-position. (C) Force acting on the loop/protein, dashed line at 65 pN indicates DNA overstretching transition. (D) Force acting on bead 2.
(TIF)

Figure S3 **Calculated position and forces acting on the scanning DNA loop for the non-sliding case.** (A) Loop x-position and (B) loop z-position. (C) Force acting on the loop/protein, dashed line at 65 pN indicates DNA overstretching transition. (D) Force acting on bead 2. Note the steep increase in forces around 2 µm trap displacement for the non-sliding case compared to the free-sliding case. The forces for the non-sliding case rise even above the DNA overstretching transition (Figure S2). The fact that experimentally events were observed at trap displacements above 2 µm strongly supports the free-sliding loop model.
(TIF)

Figure S4 **Three EcoRI proteins are detected and removed by the scanning DNA loop.** The magnetic bead deflection (red line) shows that the proteins are removed after the third scan at 20 pN applied stretching force for both DNA molecules. At t = 50 s a rebinding event is observed, but this protein is also immediately dislodged. Scans are indicated by the gray line representing the center position of the scanning DNA, calculated as the mean of the two optically trapped-bead positions.
(TIF)
