# Supplementary figures and images for: Scanning a DNA Molecule for Bound Proteins Using Hybrid Magnetic and Optical Tweezers
Source: PLoS One. 2013 Jun 3;8(6):e65329. doi: 10.1371/journal.pone.0065329 (PMC3670887; doi:10.1371/journal.pone.0065329)

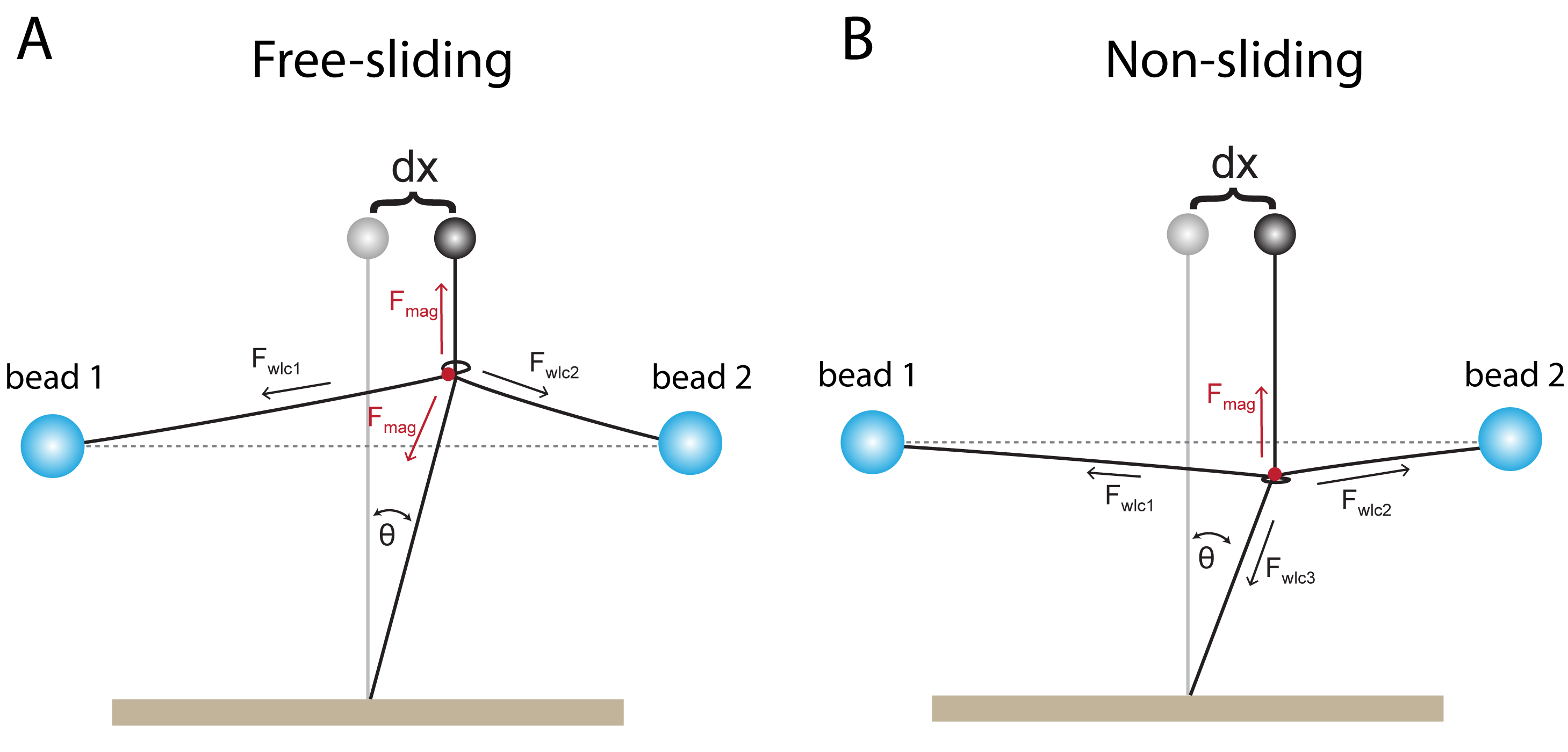

Supplement: Figure S1 — Force diagrams for the free-sliding (A) and non-sliding (B) case. Force balances at the loops shown in Figure S1 were iteratively solved using the following equations, where Fwlc was calculated using Ref. 21, with l0 the contour length of a DNA section, i.e. the contour length of a section of DNA spanning from bead to loop, lp the persistence length of DNA = 50 nm, and L the length of a DNA section. Fmag = 10 pN was the force applied on the magnetic bead and Fprot_freeSliding and Fprot_nonSliding represent the forces acting on the protein/loop for the free-sliding and non-sliding case respectively. (TIF) [file pone.0065329.s001.tif]

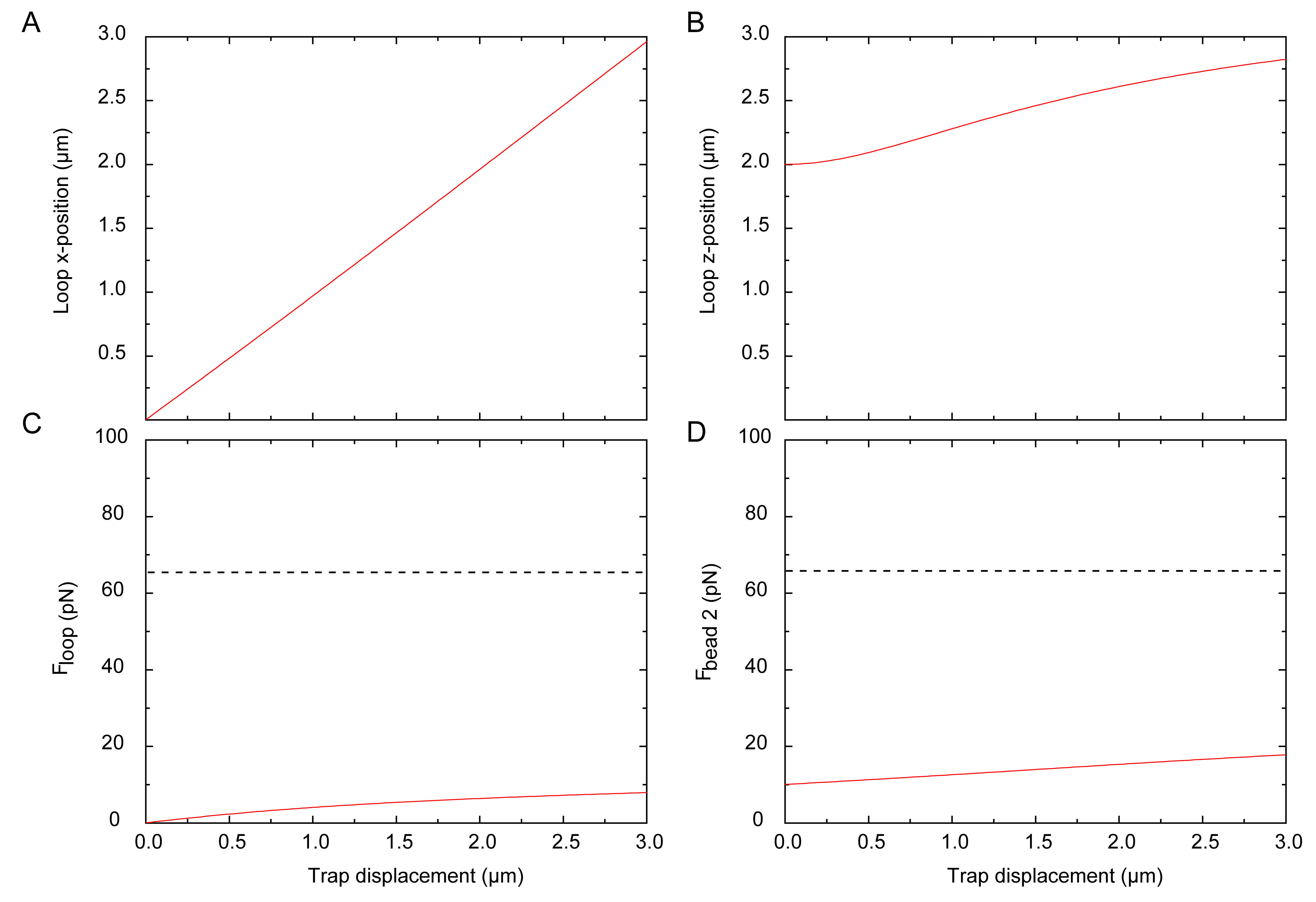

Supplement: Figure S2 — Calculated position and forces acting on the scanning DNA loop for the free-sliding case. (A) Loop x-position and (B) loop z-position. (C) Force acting on the loop/protein, dashed line at 65 pN indicates DNA overstretching transition. (D) Force acting on bead 2. (TIF) [file pone.0065329.s002.tif]

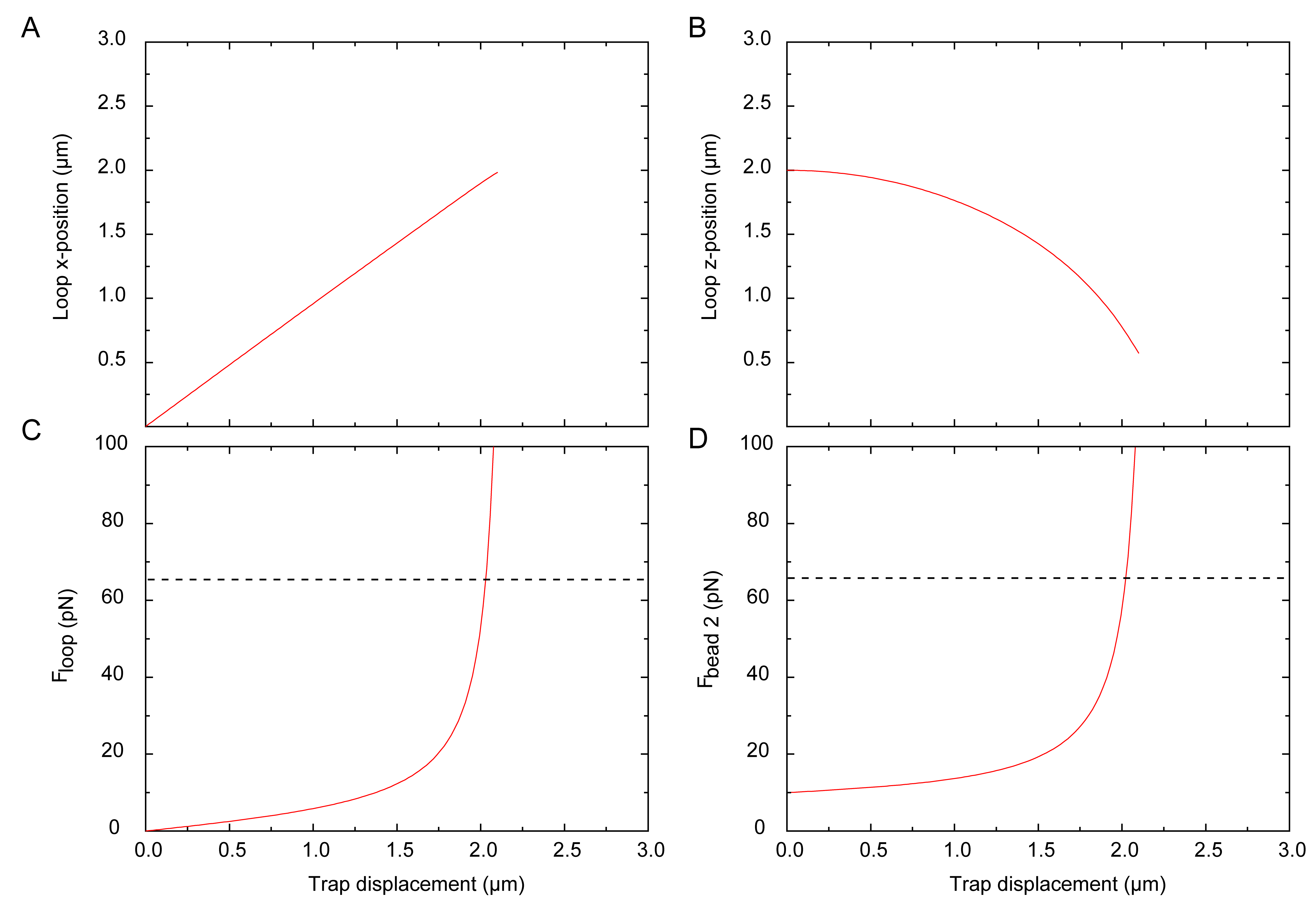

Supplement: Figure S3 — Calculated position and forces acting on the scanning DNA loop for the non-sliding case. (A) Loop x-position and (B) loop z-position. (C) Force acting on the loop/protein, dashed line at 65 pN indicates DNA overstretching transition. (D) Force acting on bead 2. Note the steep increase in forces around 2 µm trap displacement for the non-sliding case compared to the free-sliding case. The forces for the non-sliding case rise even above the DNA overstretching transition (Figure S2). The fact that experimentally events were observed at trap displacements above 2 µm strongly supports the free-sliding loop model. (TIF) [file pone.0065329.s003.tif]

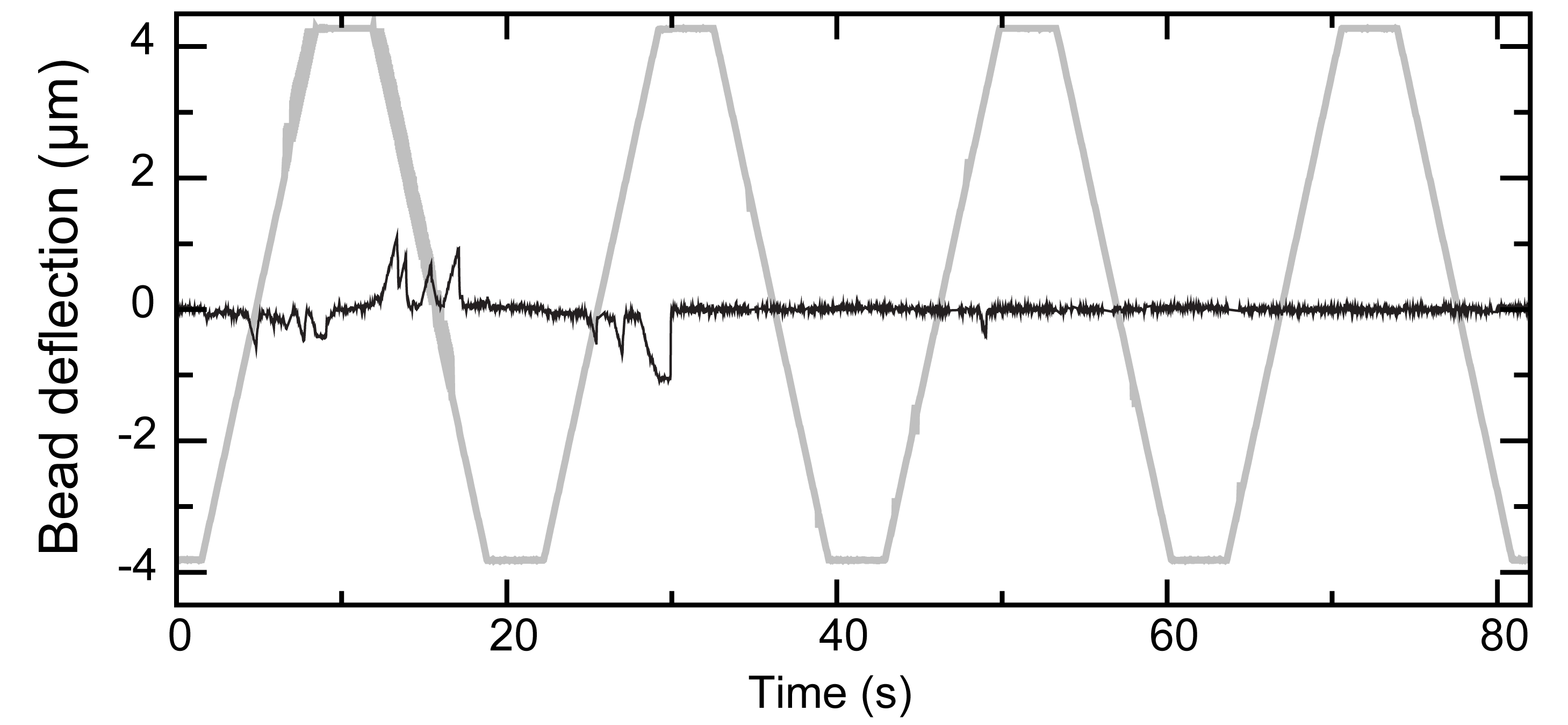

Supplement: Figure S4 — Three EcoRI proteins are detected and removed by the scanning DNA loop. The magnetic bead deflection (red line) shows that the proteins are removed after the third scan at 20 pN applied stretching force for both DNA molecules. At t = 50 s a rebinding event is observed, but this protein is also immediately dislodged. Scans are indicated by the gray line representing the center position of the scanning DNA, calculated as the mean of the two optically trapped-bead positions. (TIF) [file pone.0065329.s004.tif]
